# Effects of whole grain rye crisp bread for breakfast on appetite and energy intake in a subsequent meal: two randomised controlled trails with different amounts of test foods and breakfast energy content

**DOI:** 10.1186/1475-2891-13-26

**Published:** 2014-03-25

**Authors:** Tina Forsberg, Per Åman, Rikard Landberg

**Affiliations:** 1 Department of Food Science, BioCenter, Swedish University of Agricultural Sciences (SLU), Box 7051, SE-75007 Uppsala, Sweden; 2 Nutritional Epidemiology Unit, Institute of Environmental Medicine, Karolinska Insitutet, Stockholm, Sweden

**Keywords:** Crisp bread, Whole grain, Rye, Appetite, Energy density, Energy intake

## Abstract

**Background:**

Fibre-rich rye products have been shown to have superior effects on self-reported appetite compared to white wheat bread and some studies have shown lower energy intake after subsequent meal. The aim of the present study was to evaluate the effects of whole grain rye crisp bread (RB) versus refined wheat bread (WB) on appetite in two studies using different portion sizes and total energy intakes.

**Methods:**

Two randomised cross-over pre-load studies were conducted in 20 and 21 subjects, respectively. Appetite was rated by visual analogue scale (VAS) for 4 h. In both studies, participants were 39 ± 14 years old and had BMI 23 ± 3. The studies differed in terms of energy content of the breakfasts and proportion of energy from the treatment product as well as amount of test products. Differences between treatments within the two studies were evaluated using mixed models with repeated measures appropriate for cross-over designs.

**Results:**

In Study one, hunger and desire to eat were significantly lower (P < 0.05) after RB compared with WB, but there were no difference for fullness or difference in energy intake at lunch served ad libitum. In Study two, the portion size was lower than in Study one and the test product constituted a larger proportion of the breakfast. Fullness was significantly higher after RB compared with WB (P < 0.05) and hunger, desire to eat as well as energy intake at lunch were significantly lower (P < 0.05).

**Conclusions:**

Whole grain rye crisp bread caused lower self-reported hunger, higher fullness and less desire to eat compared to refined wheat bread. It also led to a lower energy intake after an *ad libitum* lunch. Results were stronger and/or more consistent when the test meal portion was smaller and accounted for a larger proportion of the total energy intake of the breakfast.

## Background

Obesity is a major health concern that results in enormous health related costs worldwide. Unhealthy diet is one of the lifestyle factors that contributes to development of obesity and associated diseases such as type 2 diabetes and cardiovascular disease and diet is therefore an important target for improved health [1-3]. Food intake is partly regulated by feelings of hunger and satiety and food characteristics such as energy-density, macronutrient composition, food structure and sensory attributes modulate these feelings [4]. Hunger feelings are major reasons for failed weight-loss attempts and developing foods that affect hunger and satiety in a beneficial direction could contribute to weight management [5]. Consumption of foods that cause higher satiety per calorie may lead to lower eating motivations during the day and to subsequently lower energy intake. Many studies, but not all, have shown that dietary fibre and whole grain foods may promote feelings of satiety and reduce hunger in short term [6]. The effects are ascribed to increased viscosity and gel formation caused by certain soluble fiber which affects gastric distension and emptying as well as nutrient absorption rate [7,8] which in turn could affect different stages in the ‘satiety cascade’ [9]. Fiber-rich whole grain foods may also affect secretion of satiety-related hormones [10] and gut fermentation may affect satiety via different pathways, including effects on hormones [11,12].

The relationship between self-reported satiety and hunger and food/energy intake is not straight forward [4] and other factors also affect body weight management. However, a recent meta-analysis including 23 studies concluded that a 15% change in self-reported satiety on a VAS-scale was associated with significant change in food intake [13]. In total, about 25 short-term studies have been published on the short-term post-meal satiety after intake of 60 different fiber-rich cereal food products [14]. Many of the studies reported increased satiety, suppressed hunger and/or a decreased in energy intake in a subsequent meal after consumption of foods rich in fiber or whole grain compared with iso-caloric portions of low-fiber control foods [14].

Among cereal foods, fibre-rich rye products have consistently shown beneficial effects on glucose metabolism and self-reported satiety, suppression of hunger and/or energy intake in a subsequent meal [15-21]. These studies have included breads and porridges based on intact or milled rye fractions, representing large differences in food structure, which is known to affect metabolism, glycemic index and satiety [16]. However, so far no study has investigated the effect of whole grain rye crisp bread, which is an important contributor to whole grain rye intake in Sweden [22]. Crisp bread contains only about 5-8% water and has a different microstructure than porridge and soft bread. Under iso-caloric, ready-to-eat conditions, lower amount of crisp bread than soft bread or porridge therefore is needed to provide equal energy. The aim of the present study was to investigate short term effects on satiety, hunger and desire to eat as well as energy intake in a subsequent meal after a normal breakfast with iso-caloric rye crisp bread (RB) or refined wheat bread (WB). Because there is no common practice regarding amounts used in pre-load studies [4], we evaluated the effects in two separate randomized controlled meal studies where two different amounts of test products and breakfast energy contents were tested.

## Materials and methods

### Study design and subjects

In both studies, subjects followed a randomised, balanced, cross-over design, in which all participants had both test breakfasts in random order. Study days were separated by a wash-out period of six days to avoid carry-over effects. Study two was conducted six months after Study one.

In both studies, 24 healthy men and women were recruited through advertisement in a local newspapers and by sending e-mails to employees at the the BioCentre at Swedish University of Agricultural Sciences, Uppsala, Sweden. Only those who regularly consumed breakfast, lunch and dinner and who did not follow any diets including vegetarians and vegans were allowed to participate. Additional exclusion criteria included dieting, use of tobacco, BMI < 18 or > 30, physiological or psychological problems with eating, gastrointestinal problems or other medical conditions that were likely to affect appetite or food intake including food intolerances or allergies. Women that were pregnant, lactating or wishing to become pregnant during the study period were also excluded. Persons aged over 65 were excluded as high age can result in effects on appetite and a decreased food intake. Written informed consent was obtained from each participant. The study was carried out in compliance with the Helsinki Declaration. Appetite ratings and anthropometric data was coded and could not be traced to any individual. Due to complete blinding and because no invasive methods were used, the local ethical committee in Uppsala judged no ethical application was needed. Written informed consent for participation in the study was obtained from all participants.

### Appetite ratings and energy intake after ad libitum lunch

Study participants were instructed by e-mail to abstain from consuming any food or beverage after 20.00 the day preceding the first study day and the last meal should not contain any products rich in fibre such as whole grain foods, fruit and vegetables. They were also instructed to not perform any vigorous physical exercise during the 24 hours before the study occasion. If these criteria were slightly violated, participants were instructed to repeat the same activity before the next study occasion to minimise any confounding effects.

In the morning of the test day, participants were instructed to come fasted to the study premises at Ultuna Campus, Swedish University of Agricultural Sciences. During fasting subjects were asked to refrain from water intake at least 2 hours before the study. Upon arrival, participants were weighted (with 0.01 kg precision), height was measured (0.5 cm precision) and they were asked about their date of birth, last meal and drink. Furthermore, participants were provided with a hand-held computer, model z22 (Palm Inc, Sunnyvale, USA) and instructed on how to use the electronic VAS to score their feelings of hunger, fullness and desire to eat. Participants were seated together at round tables in two separate rooms according to treatment.

Before breakfast, subjects filled out appetite ratings on the Palm computer. Subjects then had their breakfast between 8.00 and 9.00 and were asked to finish the meal within 15 minutes. Data on was recorded. Subjects filled out new appetite ratings 30 minutes from the first question and then every 30 minutes until lunch. The Palm computer indicated when it was time to reply to the three questions given in a sequence: ‘How hungry do you feel right now?’ , ‘How full do you feel right now?’ and ‘How strong is you desire to eat right now?’ along with the respective scales marked at opposite ends: not at all hungry/extremely hungry, not at all full/extremely full, extremely strong/not strong at all. Like the conventional 100 mm VAS, the computer translates the mark that the participant makes along the scale to a number between 0–100. The lunch was served ad libitum exactly 4 hours after the initiation of the breakfast. Conversation was allowed with the exception of discussing the study or comparing ratings. Participants were not allowed to eat or drink anything between breakfast and lunch, but were allowed to perform their usual occupation. Information about the study was given both orally and in written to those who reported interest to participate.

In Study two, a few of the Palm computers did not work and thus scales on paper were used on which appetite was scored with a pen. Scales were measured and values were entered manually in an excel-speadsheet. The same scale as on computers were used and previous studies have shown that the methods give similar results in agreement and can be used interchangeably with a minimal bias [23-25].

### Test products and meals

The intervention product used in both studies was a whole grain rye crisp bread (RB) and the control product a refined soft wheat bread (WB) (Table 1). Dietary fibre components in the two breads were analysed by the Uppsala method [26] and mixed-linkage β-glucan [27] and fructan [28] contents by using the Megazyme kits K-BGLU and K-FRUC, respectively. The breads were served as part of a standardised breakfast together with margarine, ham, a small glass of orange juice (2 dl in Study one and 1 dl in Study two) and cheese (only served in Study one) (Table 2). Participants could choose between a cup of coffee or tea (the same amount and choice of beverage was used for both RB and WB treatments). Energy content was adjusted by adding margarine to make RB and WB treatments isocaloric. Study one and two differed in four important aspects; total fibre content and composition, total energy content, amount test products and the proportion of total energy content originating from the test products (Table 2). In Study one, the total energy content was about 40% more than in Study two and the test prouct amount and energy was 20% lower in Study two. In Study one, 48% of the energy content of the treatment breakfast originated from the breads, whereas in Study two, 61% of the total energy content originated from the breads.

**Table 1:** Dietary fibre content and composition in whole grain rye crisp bread (RB) and refined wheat bread (WB)

	**Treatment bread**	
**Fibre**	**RB**	**WB**
Total dietary fibre^a^	17.5	6.4
Arabinoxylan^b^	8.2	2.1
β-Glucan	1.9	0.27
Cellulose and resistant starch^c^	2.4	2.3
Fructan	2.9	0.38

^a^Calculated as the sum of dietary fibre components analysed by the Uppsala method [26] and fructan content [28].

^b^Calculated as sum of arabinose and xylose residues analysed by the Uppsala method [26].

^c^Calculated as the difference between glucose residues analysed by the Uppsala method [26] and total β-glucan [27].

Values are given as% of dry matter.

**Table 2:** The amount, macro-nutrient and energy content of the breakfasts provided in Study one (S1) and two (S2)

	**Amount *(g)***		**Nutrient**								**Energy *(kJ)***	
			**Fat *(g)***		**Protein *(g)***		**Carbohydrate *(g)***		**Dietary fibre *(g)***			
	**S1**	**S2**	**S1**	**S2**	**S1**	**S2**	**S1**	**S2**	**S1**	**S2**	**S1**	**S2**
**RB**												
Whole grain rye crisp bread	80	64	1.9	1.5	7.7	6.1	52	42	13	10	1188	953
Margarine	30	20	11.7	7.8	0.1	0.1	0.9	0.6	0	0	450	301
Ham	27	30	0.8	0.9	5.9	6.6	0.3	0.3	0	0	136	151
Cheese	25	-	7	-	5.6	-	0	-	0	-	357	-
Orange juice	200	100	0.2	0.1	1.4	0.7	18	9.0	1.4	0.7	348	174
Total	362	214	22	10	21	14	71	52	14	11	2479	1573
E%			33	24	14	15	48	56	5	6	100	100
**WB**												
Refined soft wheat bread	108	86	3.8	3.0	9.7	6.9	50	40	3.8	2.6	1180	936
Margarine	30	20	11.7	7.8	0.1	0.1	0.9	0.6	0	0	450	301
Ham	27	30	0.8	0.9	5.9	6.6	0.3	0.3	0	0	136	152
Cheese	25	-	7		5.8		0	-	0	-	357	-
Orange juice	200	100	0.2	0.1	1.4	0.7	18	9.0	1.4	0.7	348	174
Total	390	241	24	12	23	14	69	50	5.2	3.3	2475	1561
E%			35	28	13	16	47	55	2	2	100	100

Four hours after intake of breakfast, participants were served a standardised lunch consisting of the Swedish dish ‘Pyttipanna’ which is a mixture of finely chopped pieces of pork, beef, potato and onion, that had been commercially manufactured and then fried in 5 g of rape seed oil per 1000 g before serving. All participants were served a plate with 600 g of ‘Pyttipanna’ (709 kJ/100 g) and a glass with 90 g of pickled beetroot (200 kJ/100 g), a glass of water (200 mL) and an empty plate. They were instructed to eat an *ad libitum* amount of food until they felt pleasantly full. The leftovers of each participant were weighted in order to measure the amount of food they had ingested and calculate the energy intake. This procedure was the same in both studies.

### Statistical analysis

Statistical analyses were performed using SAS (version 9.2, SAS Institute Inc., USA). The level of significance was set at P < 0.05. Normality of the data was evaluated using Shapiro-Wilk’s test as well as graphically by plotting the residual variance when fitted to a linear model. When the data showed a non-Gaussian distribution, data were log-transformed. Criteria for choosing a transformed or a non-transformed model were primarily based on the graphic test for normality and secondarily on Shapiro-Wilk’s test. Effects on appetite was analysed based on VAS scores as well as for the calculated AUC for the three questions respectively. AUCs were calculated using the trapezoidal rule. Data from Study one and two was analysed separately by mixed models appropriate for cross-over designs. For VAS scores, the model included occasion, treatment, time point and, time point × treatment, occasion × treatment as fixed factors. Subject was entered as a random factor. For AUC and total energy intake after ad lib lunch, similar models were used but the factor time point was excluded. Differences in appetite between breakfasts measured with AUC as well as differences in subsequent meal energy intake were compared using adjusted mean values (least mean squares, LSM). When log-transformed data was used, the LSM presented represent the geometric means. All mean values presented for AUC and energy intakes are LSM ± standard error of the mean (SEM). Other mean values are aritmetric mean values ± standard deviation (SD). The number of participants needed to detect a 10% difference in appetite scores between the treatments with paired design and with α < 0.05 and power = 80% has previously been estimated to 13–18. Less than 8 are required when evaluating mean ratings during 4.5 h [29].

## Results

Twenty one and twenty participants fulfilled the inclusion criteria and completed Study one and two, respectively. Subjects had similar characteristics in both studies, but the number of female participants was higher in Study two (Table 3). All participants finished their breakfasts within 15 minutes in both studies. The breads used contained large differences in dietary fibre content and composition. RB contained 17.5% total dietary fibre with arabinoxylan, fructan, cellulose and resistant starch, and β-glucan as major constituents. WB had considerably lower total dietary fibre content (6.4%) with cellulose and resistant starch and arabinoxylan as major components. The wheat bread process favour formation of resistant starch compared to the rye crisp process used which is the reason for the relatively high content of cellulose and resistant starch content in WB.

**Table 3:** Characteristics of participants in Study one (S1) and two (S2)

	**Men**		**Women**		**All**	
	**S1**	**S2**	**S1**	**S2**	**S1**	**S2**
Participants (n)	10	6	11	14	21	20
Age (years)	34 ± 11	38 ± 13	44 ± 15	40 ± 15	39 ± 14	39 ± 14
Weight (kg)	78 ± 12	75 ± 15	62 ± 5	62 ± 6	70 ± 12	66 ± 12
Height (m)	1.80 ± 0.08	1.79 ± 0.07	1.66 ± 0.05	1.66 ± 0.04	1.73 ± 0.1	1.70 ± 0.08
BMI (kg/m^2^)	24 ± 4	23 ± 4	23 ± 2	22 ± 2	23 ± 3	23 ± 3

### Study one

**Figure 1:**
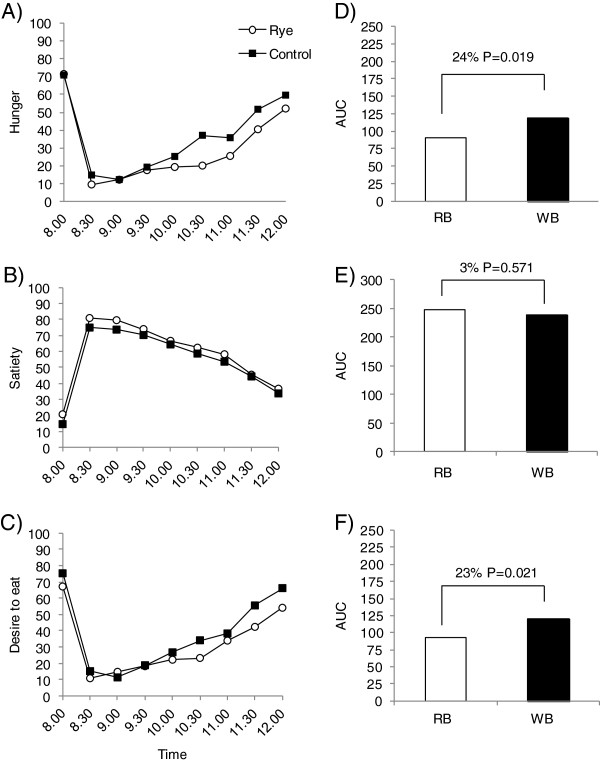
**Self-reported appetite ratings from 8.00 – 12.00 and the corresponding area under the curve (AUC) in Study one.** Self-reported **A)** hunger, **B)** satiety, **C)** desire to heat, **D)** AUC for hunger, **E)** AUC for satiety, **F)** AUC for desire to eat. Estimates in A-C represent arithmetic mean values for each treatment. The difference between rye bread (RB) and wheat bread (WB) treatments is provided as a % difference between the least square means (LSM) for each treatment AUC, estimated by mixed linear models appropriate for cross-over designs (see statistical analysis section). P < 0.05 was considered statistically significant.

Before breakfast, there were no significant difference in mean ratings for hunger, satiety and desire to eat between treatments (P > 0.05). After breakfast, participants felt less hungry after eating the RB breakfast compared to the WB breakfast ( P < 0.0001, Figure 1A, D). The differences in hunger when evaluated by comparing AUC was 24% (P = 0.02) (Figure 1D). Moreover, participants felt less hunger, regardless of breakfast type, in second treatment period (P = 0.003). An over- all difference in satiety was detected between the breakfasts (P = 0.03) (Figure 1B) but not when comparing AUCs between treatments (P = 0.571) (Figure 1E). Participants felt less desire to eat after the RB breakfast compared to the WB breakfast (P < 0.001) (Figure 1C) and in the second period, independent of treatment (P = 0.0006). The desire to eat was 23% lower (P = 0.02) after eating the RB breakfast compared to the WB breakfast when comparing the AUCs between treatments (Figure 1F).

There was no difference in energy intake between treatments after the *ad libitum* lunch exactly 4 h after the breakfast meal (P = 0.413).

### Study two

**Figure 2:**
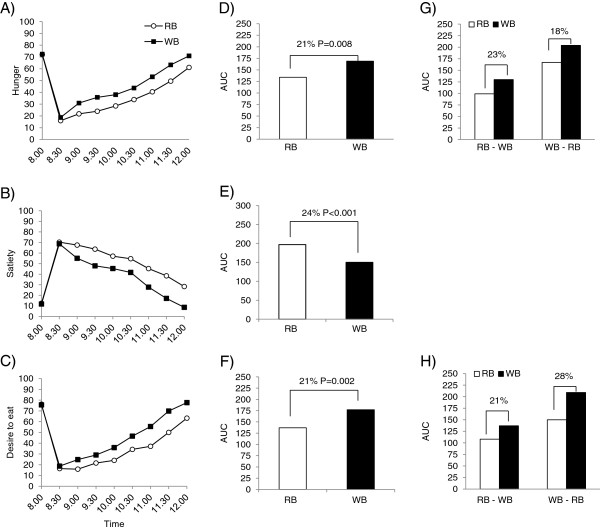
**Self-reported appetite ratings from 8.00 – 12.00 and the corresponding area under the curve (AUC) in Study 2.** Self-reported **A)** hunger, **B)** satiety, **C)** desire to heat, **D)** AUC for hunger, **E)** AUC for satiety, **F)** AUC for desire to eat. Estimates in A-C represent arithmetic mean values for each treatment. The difference between rye bread (RB) and wheat bread (WB) treatments is provided as a % difference between the least square means (LSM) for each treatment AUC, estimated by mixed linear models appropriate for cross-over designs (see statistical analysis section). P < 0.05 was considered statistically significant. When a statistically significant interaction between treatment and occasion was observed i.e. when effect of treatment appeared to be treatment order, treatment effects were evaluated separately according to treatment sequence (RB-WB or WB-RB, respectively). This was the case for **G)** hunger and **H)** desire to eat.

Before breakfast, there were no differences between the treatments (P > 0.05). After breakfast, participants were less hungry after eating the RB breakfast compared to the WB breakfast (P < 0.0001) (Figure 2A). There was also an interaction between treatment and occasion (P = 0.012), indicating that hunger scores were affected by the treatment order. The treatment order appeared to affect the magnitude of difference between breakfasts, but not the direction; participants were less hungry after eating the RB breakfast, but the hunger scores were lower in the group who started with RB and then received WB than in the group with opposite order and the difference was large enough to result in a statistically significant interaction. When hunger was evaluated based on the AUC, there was also an interaction between treatment and occasion (P = 0.006), with a slightly larger difference in hunger for the group who started with the RB (23%) compared with the group who started with the WB (18%) (Figure 2G).

Participants felt more full (P < 0.0001) after eating the RB breakfast compared to the WB breakfast (Figure 2B). The difference was 24% when comparing AUCs between treatments (P < 0.0001) (Figure 2E). Desire to eat was lower after eating the RB breakfast compared to the WB breakfast (P < 0.0001) (Figure 2C). However, there was an interaction (P = 0.028) between treatment and occasion, showing that the effect was dependent on the order in which RB or WB breakfast was served to the participants. In the group who started with RB, the difference between the breakfasts in desire to eat was less clear than for the group who started with WB. As expected, an effect of the interaction between treatment and occasion (P = 0.044) was also found for the evaluation of differences in AUC, meaning that the difference in desire to eat was statistically different between the group that received the RB and WB breakfast first, respectively. This difference between treatments was largest (28%) among participants who started with WB compared to 21% in the group who started with RB (Figure 2H).

Energy intake was about 8% lower at the *ad libitum* lunch exactly 4 h after RB compared to WB breakfast (P = 0.024). There was also an effect of occasion (P = 0.01) showing that participants had higher energy intake after the second treatment, irrespective of breakfast.

## Discussion

The present study investigated for the first time how whole grain crisp bread instead of refined wheat bread for breakfast affected the self-reported hunger, satiety and desire to eat as well as the energy intake after a subsequent meal served *ad libitum* 4 h after breakfast. Effects were evaluated in two studies where the amount of test foods as well as the total energy intake of the breakfasts differed.

In Study one, where test bread intake as well as total energy intake was relatively high, participants felt less hungry and less desire to eat after consuming RB instead of WB for breakfast. However, no statistically significant effect on fullness or in energy intake after a subsequent lunch was observed. The bread portion together with the additional breakfast foods comprised what would be considered a large breakfast (2480 kJ) [30]. Due to the large surface area of crisp bread, more spread, ham and/or cheese was needed than for a typical soft bread of similar weight.

The amount of breakfast product and the total energy intake was reduced in Study two, in order to investigate whether more consistent effects on appetite and subsequent meal energy intake could be obtained at a somewhat lower intake of product and lower total energy intake. The difference between treatments in Study two was similar in magnitude (20-28%) as in Study one, but more consistent as a statistically significant difference in self-rated satiety was also found.

Although the amount of test bread used in Study two was 20% lower than in Study one, the proportion of energy from test bread of entire breakfast was higher since the total energy of the entire breakfast was considerably reduced. The total energy in Study two was in the lower range (1580 kJ) of what would be considered a normal breakfast [31]. We speculate that the less consistent results in Study one compared with Study two were due to a too high over-all breakfast intake and/or energy intake which masks differences between tested breads due to the fact that participants reported high satiety ratings for both diets. This is to some degree supported by results from previous studies [15,16]. However, the macronutrient composition differed slightly between our our two studies and that could also have affected the results.

The finding that whole grain rye crisp bread affects satiety, hunger and desire to eat as well as energy intake in a subsequent meal, in a favorable direction is in line with previous studies with fibre-rich rye products in a similar study design. Isaksson et al. have for example consistently shown that whole grain rye porridge based on intact kernels, different milling fractions and flour or flakes, increase satiety and decrease hunger during 4 h after intake, compared with iso-caloric refined wheat bread served with identical breakfast foods [16,17,31]. Also iso-caloric rye breads containing different bran fractions, whole grain, and sifted rye flour, all showed significantly higher satiety ratings than refined wheat bread although the largest effects were observed when replacing 25-60% of the refined wheat flour for rye bran [15]. Furthermore, Rosén et al. [20] found similar effects on appetite ratings for different rye breads for breakfast compared to a refined wheat bread breakfast and they also found 16% lower energy intake at lunch when participants consumed rye kernel bread compared with refined wheat bread. These findings are in line with the 8% lower energy intake at lunch after RB compared to WB breakfast in present Study two.

In our two studies, we found no significant difference between the two test breads at the first time point (30 min) after breakfast. This is in contrast to what has been found in the studies with rye bread and porridges by Isaksson et al. [15,31], where differences between treatments started to appear immediately after intake. This difference between studies probably reflect the higher water content in soft bread and porridge which results in a larger volume compared to crisp bread. Added water incorporated into foods has been shown to increase satiety and decrease spontaneous food intake in short-term [32]. Isaksson et al. showed that the immediate effect of large difference in weight and volume caused by water between a milled kernel porridge and a kernel porridge, disappeared during the first hour after a meal [16].

In the current study we only evaluated effects up to 4 hours. In previous studies, prolonged satiety between 4.5-8 h after breakfast has been observed after consumption of rye porridge with intact kernels, but not milled kernels, probably as a result of reduced small intestinal digestion and absorption due to a physical barrier to digestive enzymes and hence, a greater extent of nutrient and dietary fibre reached the colon where they could be fermented [16]. However, high-fibre breads based on milled rye bran and whole grain flour also showed significantly higher degree of dry matter, ash, protein, fat, amylase-available starch and dietary fibre escaping small intestine digestion and in an ileostomy model [33]. This suggests that rye products based on milled flour, may also lead to a greater availability of fermentable nutrients in the colon, which could affect satiety and appetite. RB contained more fermentable fructans and arabinoxylans than the WB. It remains to test whether whole grain crisp bread also results in significant afternoon effects.

We did not assess the mechanisms underlying the beneficial effects of replacing WB for RB in the present study. However, it is interesting that RB, which has a smaller volume and different microstructure than soft bread or porridge resulted in similar effects on self-reported appetite ratings and reduction in energy intake [15-18,20,31]. This suggests that features of dietary fibre and or bioactive compounds of rye may be more important for the short-term effect (≤ 4 h) on self-rated appetite and energy intake than for example test product volume. The dietary fiber intake in the present study was higher or similar compared with previous studies using high-fiber rye products [15-18,20,31]. Positive effects on appetite derived from rye fiber may include bulking effects resulting in increased extension of the stomach and delayed gastric emptying (for viscous fiber) which in turn may affect nutrient absorption kinetics. High-fibre rye compared to refined wheat has been shown to lower postprandial insulin secretion [19,21]. This together with other early signals of satiation may have contributed to positive effects appetite ratings in the present study. The impact of bioactive compounds such as benzoxazinoids and phenolic acids on glycemic response appetite has been investigated recently, but needs to be studied further to reach a conclusion [18,34].

Taken together, results from the studies clearly showed beneficial effects of eating whole grain rye crisp bread compared with refined wheat bread as part of a normal breakfast on hunger, desire to eat and/or satiety. The difference was about 20-30% between treatments and the effects are most consistent when RB constituted a large proportion of the energy content of a small breakfast.

## Abbreviations

AUC: Area under the curve; BMI: Body mass index; RB: Whole grain rye crisp bread; VAS: Visual analogue scale; WB: Refined wheat bread (WB).

## Competing interests

RL holds a research grant from The Swedish Association of Crispbread Manufacturers (Föreningen Sveriges Spisbrödsfabrikanter) for the financing of the current study. RL did not get salary or any other financial compensation for conducting the present study or for any other purposes. No other financial competing interests exist.

## Authors’ contributions

RL and PÅ conceived and designed the study. RL conducted the trails, prepared datasets, conducted statistical analysis, interpreted data, wrote the manuscript and has the responsibility for the final content. TF assisted in the conduct of the second trail and conducted statistical analysis under the supervision of RL and contributed to data interpretation. All authors read and approved the final manuscript.
